# Modelling, Simulation and Dynamic Sliding Mode Control of a MEMS Gyroscope

**DOI:** 10.3390/mi12020190

**Published:** 2021-02-13

**Authors:** Yunmei Fang, Wen Fu, Cuicui An, Zhuli Yuan, Juntao Fei

**Affiliations:** 1 College of Mechanical and Electrical Engineering, Hohai University, Changzhou 213022, China; yunmeif@163.com (Y.F.); wenfu2021@163.com (W.F.); cuicuian@163.com (C.A.); jackhohai@126.com (Z.Y.); 2 Jiangsu Key Laboratory of Power Transmission and Distribution Equipment Technology, Changzhou 213022, China

**Keywords:** adaptive control, dynamic sliding mode control, backstepping control

## Abstract

An adaptive dynamic sliding mode control via a backstepping approach for a microelectro mechanical system (MEMS) vibratory z-axis gyroscope is presented in this paper. The time derivative of the control input of the dynamic sliding mode controller (DSMC) is treated as a new control variable for the augmented system which is composed of the original system and the integrator. This DSMC can transfer discontinuous terms to the first-order derivative of the control input, and effectively reduce the chattering. An adaptive dynamic sliding mode controller with the method of backstepping is derived to real-time estimate the angular velocity and the damping and stiffness coefficients and asymptotical stability of the designed systems can be guaranteed. Simulation examples are investigated to demonstrate the satisfactory performance of the proposed adaptive backstepping sliding mode control.

## 1. Introduction

Microelectro mechanical system (MEMS) gyroscopes can measure the sensor angular velocity of inertial navigation and guidance systems, widely used in aviation, aerospace, marine and positioning fields. However, parameter uncertainties and external disturbances, the manufacturing errors, and the influence of the ambient temperature decrease the accuracy and sensitivity of the micro gyroscope. The manufacturing errors and the influence of the external conditions as main factors affecting the decrease in the accuracy and sensitivity of the gyro system, the nonlinear effects in the model applied is also of great importance. The problem concerning the impact of the nonlinearity is discussed [1,2,3]. Then, compensation for manufacturing tolerances and accurate measurement of the angular velocity are the main problems of microscopes. During the past years, some new control strategies have been investigated to compensate for the performance and parameters of the MEMS gyroscopes. Park et al. [4,5] developed an adaptive trajectory-switching algorithm for a MEMS gyroscope. Batur et al. [6] developed a sliding mode controller of a simulated MEMS gyroscope. Leland et al. [7] proposed an adaptive control of a MEMS gyroscope using Lyapunov methods. Chen et al. [8] implemented an optimized double closed-loop control system for a MEMS gyroscope. Xu et al. [9] utilized a composite neural strategy with a finite time controller for a microgyscope. Adaptive sliding mode control and adaptive control with a fuzzy compensator for a MEMS gyroscope have been investigated in [10,11,12,13].

Dynamic sliding mode control (DSMC) schemes [14,15,16,17,18,19] have attracted great interest in recent years because they are special approaches to reducing the chattering through an integrator in the system. The time derivative of the control input is treated as a new control variable for the augmented system where the augmented system includes the original system and the integrator. Since no boundary layer is used in the dynamic sliding mode controller, chattering reduction can be obtained by using an integrator and the property of perfect disturbance rejection can be guaranteed. Zhao [20] proposed adaptive backstepping sliding mode control for leader-follower multi-agent systems. Lin et al. [21] studied adaptive backstepping sliding mode control for linear induction motor drive. Lin et al. [22] proposed a Field Programmable Gate Array (FPGA)-based adaptive backstepping sliding-mode controller for linear induction motor drive. Ansarifar et al. [23] proposed an adaptive DSMC method for non-minimum phase systems. Sousy et al. [24] developed an adaptive DSMC system with recurrent Radial-Basis Function Networks (RBFN) for an induction motor servo drive. Neural control and fuzzy control have the capacity to approximate unknown smooth functions and have been widely used in identification and control [25,26,27,28]. 

However, an adaptive backstepping scheme combined with dynamic sliding mode controller has not been applied to a MEMS gyroscope yet. The backstepping method is a powerful design tool for dynamic systems with pure or strict feedback forms. The gyroscope equations can be transformed into an analogically cascade system that is easily implemented by the backstepping method. This work is an extended version of the 2013 work [18] and the new contributions are the backstepping scheme is combined with the adaptive dynamical sliding mode controller to improve the robustness, and estimate the system parameters and angular velocity.

In this paper, an adaptive dynamic sliding mode controller based on backstepping control is designed to realize position tracking and effectively decrease the chattering problem. The advantages of the proposed controller can be summarized as follows:

(1) Adaptive control, DSMC and backstepping control are combined and applied to a MEMS gyroscope. DSMC using the derivative of the switching function is utilized to eliminate the chattering and attenuate the model uncertainties and external disturbances and adaptive control is derived to estimate the dynamics of the micro gyroscope. Hence, dynamic sliding mode control not only removes some of the fundamental limitations of the traditional approach but also provides improved tracking accuracy under sliding mode.

(2) The proposed DSMC adds additional compensators to achieve system stability, thereby obtaining the desired system property. An integrator is added in the front end to transform the original system into an augmented system, with the derivative of the original control input as the system input. Therefore, the proposed integrator can filter out high frequency noise.

(3) The advantages of the backstepping design are that it is able to relax the matching condition and avoid cancelation of useful nonlinearities. The procedure of backstepping design is to develop a controller recursively by regarding some of the state variables as “virtual controls” and deriving control laws to improve the robustness.

The paper is organized as follows. In Section 2, the dynamics of the MEMS vibratory gyroscope are established. In Section 3, an adaptive dynamic sliding mode controller based on backstepping method is developed. Simulation studies are given in Section 4 to prove the performance. Conclusions are provided in Section 5.

## 2. Dynamic Model of MEMS Gyroscope

**Figure 1:**
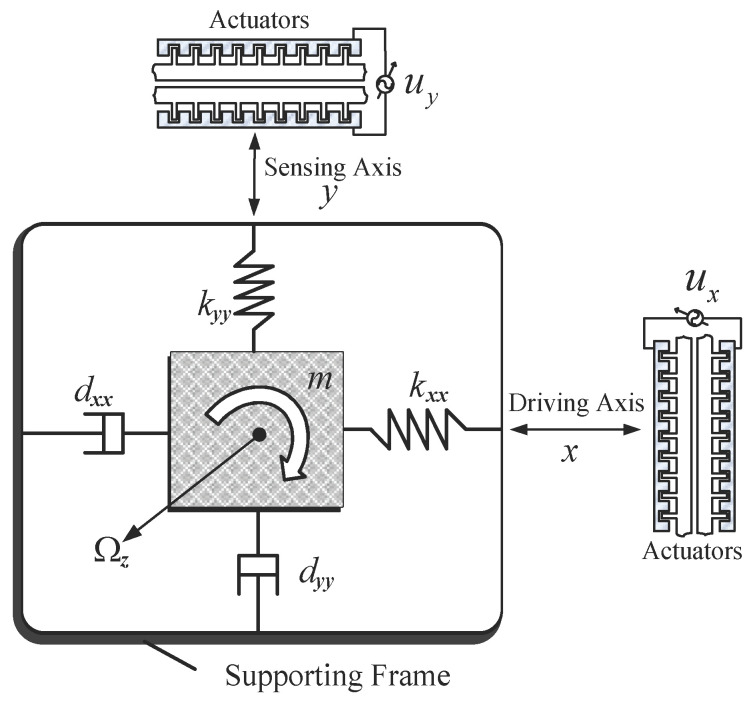
Schematic diagram of a microelectro mechanical system (MEMS) gyroscope in the *x-y* plane.

The typical MEMS vibratory gyroscope depicted in Figure 1 has a proof mass suspended by springs, an electrostatic actuation, and sensing mechanisms that can force an oscillatory motion and sense the position and velocity of the proof mass.

We assume that the table where the proof mass is mounted is moving with a constant velocity; the gyroscope is rotating at a constant angular velocity $\Omega_{z}$ over a sufficiently long time interval. Since the angular rate is usually small compared to the natural frequency of the system and the proof mass is also small, the centrifugal forces $m\Omega_{z}^{2}x$, $m\Omega_{z}^{2}y$, are assumed to be negligible or absorbed as part of the spring terms as unknown variations; the gyroscope undergoes rotation about the z axis only, and thereby Coriolis force acting on the plane perpendicular to *z* axis.

Referring to [5], with these assumptions, the dynamics of the gyroscope become

(1) $$\begin{array}{l} m\ddot{x}+d_{xx}\dot{x}+d_{xy}\dot{y}+k_{xx}x+k_{xy}y=u_{x}+2m\Omega_{z}\dot{y}\\ m\ddot{y}+d_{xy}\dot{x}+d_{yy}\dot{y}+k_{xy}x+k_{yy}y=u_{y}-2m\Omega_{z}\dot{x} \end{array}$$

Fabrication imperfections result in the asymmetric spring and damping terms, $k_{xy}$ and $d_{xy}$. The spring and damping terms, $k_{xx}$, $k_{yy}$, $d_{xx}$, and $d_{yy}$ in the x and y axes are mostly known, but have small unknown variations from their nominal values. The proof mass can be determined very accurately, and $u_{x}$, $u_{y}$ are the electrostatic forces in the x and y directions.

Define non-dimensional time $t^{*}=t/t_{0}=\omega_{0}t$; $t_{0}$ is reference time, $\omega_{0}=1/t_{0}$ is resonance frequency. Define non-dimensional position $q^{*}=q/q_{0}$, $q={\left[\begin{array}{cc} x & y \end{array}\right]}^{T}$; $q_{0}$ is reference position

(2) $${\dot{q}}^{*}=\frac{dq^{*}}{dt^{*}}=\left(\frac{1}{q_{0}\omega_{0}}\right)\dot{q},\;{\ddot{q}}^{*}=\frac{d^{2}q^{*}}{dt^{*2}}=\left(\frac{1}{q_{0}\omega_{0}^{2}}\right)\ddot{q}$$

On both sides of the Equation (1) divide by the mass m, reference position $q_{0}$, the square of the resonance frequency $w_{0}^{2}$, so we can obtain

(3) $$\begin{array}{l} {\ddot{x}}^{*}+{d_{xx}}^{*}{\dot{x}}^{*}+{d_{xy}}^{*}{\dot{y}}^{*}+{\omega_{x}}^{2}x^{*}+\omega_{xy}y^{*}={u_{x}}^{*}+2{\Omega_{z}}^{*}{\dot{y}}^{*}\\ {\ddot{y}}^{*}+{d_{xy}}^{*}{\dot{x}}^{*}+{d_{yy}}^{*}{\dot{y}}^{*}+\omega_{xy}x^{*}+{\omega_{y}}^{2}y^{*}={u_{y}}^{*}-2{\Omega_{z}}^{*}{\dot{x}}^{*} \end{array}$$

where

$$\begin{array}{l} \frac{d_{xx}}{m\omega_{0}}\to {d_{xx}}^{*},\frac{d_{xy}}{m\omega_{0}}\to {d_{xy}}^{*},\frac{d_{yy}}{m\omega_{0}}\to {d_{yy}}^{*},\\ \frac{u_{x}}{m{\omega_{0}}^{2}q_{0}}\to {u_{x}}^{*},\frac{u_{y}}{m{\omega_{0}}^{2}q_{0}}\to {u_{y}}^{*},\\ \frac{k_{xx}}{m{\omega_{0}}^{2}}\to \omega_{x}^{2},\frac{k_{xy}}{m{\omega_{0}}^{2}}\to \omega_{xy},\frac{k_{yy}}{m{\omega_{0}}^{2}}\to \omega_{y}^{2},\frac{\Omega_{z}}{\omega_{0}}\to {\Omega_{z}}^{*} \end{array}$$

Equation (3) is a mathematical model of the MEMS gyroscope under ideal conditions. Considering the presence of model uncertainties and external disturbances of a MEMS gyroscope under the actual conditions, ignoring the superstar for the convenience of notation, then rewriting non-dimensional model (3) in matrix form yields

(4) $$\{\begin{array}{c} {\dot{x}}_{1}=x_{2}\\ {\dot{x}}_{2}=f(x,y)\theta +u+d, \end{array}$$

where $x_{1}=q,x_{2}=\dot{q}$, $f(x,y)=-\left[\begin{array}{ccccccc} \dot{x} & \dot{y} & 0 & -2\dot{y} & x & y & 0\\ 0 & \dot{x} & \dot{y} & 2\dot{x} & 0 & x & y \end{array}\right]$, θ is the parameter of a MEMS gyroscope as $\theta ={\left[\begin{array}{ccccccc} d_{xx} & d_{xy} & d_{yy} & \Omega_{Z} & w_{x}^{2} & w_{xy} & w_{y}^{2} \end{array}\right]}^{T}$, d is the model uncertainties and external disturbances of a MEMS gyroscope. We assume that the input disturbances d and their derivative $\dot{d}$ are bounded signals.

Suppose an ideal oscillator generates a reference trajectory and the control objective is to make the trajectory of the MEMS gyroscope follow that of the reference model. The reference model is defined as

(5) $$\ddot{r}+K_{m}r=0,$$

where r is the reference trajectory vector, $K_{m}=diag\left\{\begin{array}{cc} \omega_{1}^{2} & \omega_{2}^{2} \end{array}\right\}$; $\omega_{1},\;\omega_{2}$ are the ideal nature frequency of the reference trajectory in the x and y directions.

The tracking error is defined as

(6) $$\{\begin{array}{c} e_{1}=x_{1}-r\\ e_{2}=x_{2}-\alpha , \end{array}$$

where α is a virtual controller.

The virtual controller is defined as

(7) $$\alpha =-c_{1}e_{1}+\dot{r},$$

where the parameter of virtual controller $c_{1}>0$. So, the time derivative of the α is

(8) $$\begin{array}{cc} \dot{\alpha } & =-c_{1}{\dot{e}}_{1}+\ddot{r}=-c_{1}({\dot{x}}_{1}-\dot{r})+\ddot{r}\\ & =-c_{1}(x_{2}-\dot{r})+\ddot{r}=-c_{1}(e_{2}+\alpha -\dot{r})+\ddot{r}\\ & =-c_{1}(e_{2}-c_{1}e_{1}+\dot{r}-\dot{r})+\ddot{r}\\ & =-c_{1}e_{2}+c_{1}^{2}e_{1}+\ddot{r} \end{array}$$

In the backstepping control, the introduction of virtual control is essentially a static compensation idea. The front subsystem must achieve stabilization purposes through the virtual control of the back subsystem.

## 3. Design and Stability Analysis of Dynamic Sliding Mode Controller

**Figure 2:**
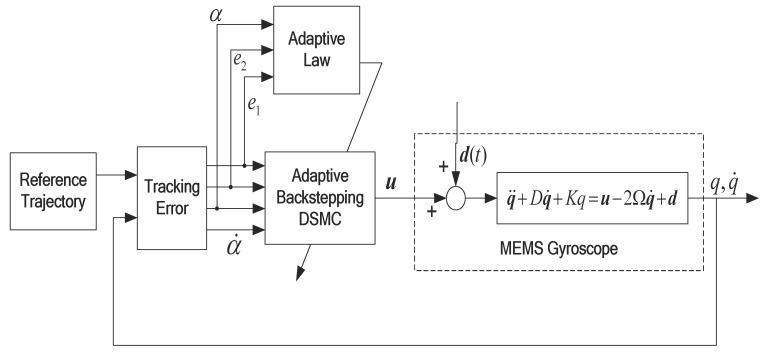
Block diagram of an indirect adaptive dynamic sliding mode controller (DSMC) based on the backstepping method.

In this section, an adaptive DSMC method based on backstepping design is developed for the trajectory tracking and system identification of a MEMS gyroscope as shown in Figure 2. The control target is to obtain real-time compensation for fabrication imperfections and identification of the system parameters and angular velocity. The backstepping dynamic sliding controller designs the time derivative of the control input and the control input obtained by integrator is proposed to control the MEMS gyroscope.

We select the first Lyapunov function as follows:

(9) $$V_{1}=\frac{1}{2}e_{1}^{T}e_{1}$$

The time derivative of the $V_{1}$ is

(10) $${\dot{V}}_{1}=e_{1}^{T}{\dot{e}}_{1}=e_{1}^{T}(x_{2}-\dot{r})=e_{1}^{T}(e_{2}+\alpha -\dot{r})=e_{1}^{T}(e_{2}-c_{1}e_{1})=-c_{1}e_{1}^{T}e_{1}+e_{1}^{T}e_{2}$$

When $e_{2}=0$, it is easy to know that ${\dot{V}}_{1}=-c_{1}e_{1}^{T}e_{1}$ meet the negative qualitative. So, the system $e_{1}=x_{1}-r$ is globally asymptotically stable and the error $e_{1}$ asymptotically converges to zero.

Define the second Lyapunov function as follows

(11) $$V_{2}=V_{1}+\frac{1}{2}e_{2}^{T}e_{2}+\frac{1}{2}s^{T}s+\frac{1}{2}{\tilde{\theta }}^{T}\tau^{-1}\tilde{\theta },$$

where $\hat{\theta }$ is a parameter estimate, $\tilde{\theta }=\theta -\hat{\theta }$ is the estimation error of the MEMS gyroscope parameter, s is the sliding surface function, and τ is an adaptive gain.

Thinking about Equations (4) and (6), the sliding surface is defined as

(12) $$s=ce_{2}+{\dot{e}}_{2}=ce_{2}+{\dot{x}}_{2}-\dot{\alpha }=ce_{2}+f(x,y)\hat{\theta }+u+d-\dot{\alpha }$$

where c is a positive definite constant to be selected.

Substituting Equation (8) into Equation (12) yields

(13) $$s=(c+c_{1})e_{2}+f(x,y)\hat{\theta }+u+d-c_{1}^{2}e_{1}-\ddot{r}$$

Referring to Equations (4) and (13), we can obtain

(14) $$\begin{array}{cc} {\dot{x}}_{2} & =s-ce_{2}-f(x,y)\hat{\theta }-d+\dot{\alpha }+f(x,y)\theta +d\\ & =s-ce_{2}+f(x,y)\tilde{\theta }+\dot{\alpha } \end{array}$$

The derivative of the sliding surface is

(15) $$\dot{s}=(c+c_{1})(f(x,y)\theta +u+d-\ddot{r})+cc_{1}(e_{2}-c_{1}e_{1})-\dddot{r}+\dot{f}(x,y)\hat{\theta }+f(x,y)\dot{\hat{\theta }}+\dot{u}+\dot{d}$$

The time derivative of the $V_{2}$ is

(16) $$\begin{array}{cc} {\dot{V}}_{2} & ={\dot{V}}_{1}+e_{2}^{T}{\dot{e}}_{2}+s^{T}\dot{s}+\tilde{\theta }\tau^{-1}\dot{\tilde{\theta }}\\ & =-c_{1}e_{1}^{T}e_{1}+e_{1}^{T}e_{2}+e_{2}^{T}({\dot{x}}_{2}-\dot{\alpha })+s^{T}\dot{s}-{\tilde{\theta }}^{T}\tau^{-1}\dot{\hat{\theta }}\\ & =-c_{1}e_{1}^{T}e_{1}+e_{1}^{T}e_{2}+e_{2}^{T}(s-ce_{2}+f(x,y)\tilde{\theta })+s^{T}\dot{s}-{\tilde{\theta }}^{T}\tau^{-1}\dot{\hat{\theta }}\\ & =-c_{1}e_{1}^{T}e_{1}+e_{1}^{T}e_{2}-ce_{2}^{T}e_{2}+s^{T}[e_{2}+(c+c_{1})(f(x,y)\theta +u+d-\ddot{r})\\ & +cc_{1}(e_{2}-c_{1}e_{1})-\dddot{r}+\dot{f}(x,y)\hat{\theta }+f(x,y)\dot{\hat{\theta }}+\dot{u}+\dot{d}]+{\tilde{\theta }}^{T}(f^{T}(x,y)e_{2}-\tau^{-1}\dot{\hat{\theta }}) \end{array}$$

To make ${\dot{V}}_{2}\leq 0$, we choose a dynamic sliding mode control law as:

(17) $$\begin{array}{ll} \dot{u}= & -[e_{2}+(c+c_{1})(f(x,y)\hat{\theta }+u-\ddot{r})+cc_{1}(e_{2}-c_{1}e_{1})-\dddot{r}+\dot{f}(x,y)\hat{\theta }\\ & \;+f(x,y)\dot{\hat{\theta }}]-\frac{s}{{\left\|s\right\|}^{2}}e_{1}^{T}e_{2}-\rho \frac{s}{\left\|s\right\|}, \end{array}$$

where ρ is a chosen positive constant.

Substituting Equation (17) into Equation (16) yields

(18) $$\begin{array}{cc} {\dot{V}}_{2} & =-c_{1}e_{1}^{T}e_{1}-ce_{2}^{T}e_{2}-\rho s^{T}\frac{s}{\left\|s\right\|}+{\tilde{\theta }}^{T}\left((c+c_{1})f^{T}(x,y)s+f^{T}(x,y)e_{2}-\tau^{-1}\dot{\hat{\theta }}\right)+s^{T}\left((c+c_{1})d+\dot{d}\right)\\ & =-c_{1}e_{1}^{T}e_{1}-ce_{2}^{T}e_{2}-\rho s^{T}\frac{s}{\left\|s\right\|}+{\tilde{\theta }}^{T}\left(f^{T}(x,y)((c+c_{1})s+e_{2})-\tau^{-1}\dot{\hat{\theta }}\right)+s^{T}\left((c+c_{1})d+\dot{d}\right) \end{array}$$

To make ${\dot{V}}_{2}\leq 0$, we choose an adaptive law

(19) $$\dot{\hat{\theta }}=\tau f^{T}(x,y)((c+c_{1})s+e_{2})$$

Substituting Equation (19) into Equation (18) yields

(20) $$\begin{array}{ll} {\dot{V}}_{2} & =-c_{1}e_{1}^{T}e_{1}-ce_{2}^{T}e_{2}-\rho s^{T}\frac{s}{\left\|s\right\|}+s^{T}\left((c+c_{1})d+\dot{d}\right)\\ & \;=-c_{1}e_{1}^{T}e_{1}-ce_{2}^{T}e_{2}-\rho \left\|s\right\|+s^{T}\left((c+c_{1})d+\dot{d}\right)\\ & \;\leq -c_{1}{\left\|e_{1}\right\|}^{2}-c{\left\|e_{2}\right\|}^{2}-\rho \left\|s\right\|+\left\|s\right\|\left((c+c_{1})\left\|d\right\|+\left\|\dot{d}\right\|\right) \end{array}$$

It is assumed that $\left\|d\right\|\leq \eta_{1},\left\|\dot{d}\right\|\leq \eta_{2}$, then Equation (20) can become the following

(21) $$\begin{array}{ll} {\dot{V}}_{2} & \leq -c_{1}{\left\|e_{1}\right\|}^{2}-c{\left\|e_{2}\right\|}^{2}-\rho \left\|s\right\|+\left\|s\right\|\left((c+c_{1})\eta_{1}+\eta_{2}\right)\\ & \;=-c_{1}{\left\|e_{1}\right\|}^{2}-c{\left\|e_{2}\right\|}^{2}-\left\|s\right\|\left(\rho -\left((c+c_{1})\eta_{1}+\eta_{2}\right)\right) \end{array}$$

With the choice of $\rho >\left((c+c_{1})\eta_{1}+\eta_{2}\right)$, ${\dot{V}}_{2}\leq 0$. ${\dot{V}}_{2}$ is a negative semi-definite mean V, s and $\tilde{\theta }$ are all bounded. $\dot{s}$ is also bounded. From Barbalat lemma, s(t) asymptotically converges to zero, $\underset{t\to \infty }{\lim}s\left(t\right)=0$, then e(t) can also converge to zero asymptotically. Therefore asymptotical stability of the designed system can be guaranteed. Thus, the method by which the adaptive dynamic sliding mode control based on the backstepping approach can adaptively control the MEMS gyroscope and reduce the chattering has been theoretically proven. The fact that the resonance frequency of the x-axis is different from that of the y-axis means that PE condition is satisfied. If reference signals are persistently excited, then adaptive law (19) guarantees that $\tilde{\theta }\to 0$ and θ converge to their true values. Thus the unknown angular velocity as well as all other system parameters can also converge to their actual values.

## 4. Simulation Study

In this section, based on the backstepping design, an adaptive DSMC strategy is designed for the trajectory tracking and system identification of the MEMS gyroscope. The parameters of the micro gyroscope sensor are described as:

$$\begin{array}{l} m=1.8\times 10^{-7}\;\mathrm{kg},k_{xx}=63.955\;N/m,k_{yy}=95.92\;N/m,k_{xy}=12.779\;N/m\\ d_{xx}=1.8\times 10^{-6}\;N\cdot s/m,d_{yy}=1.8\times 10^{-6}\;N\cdot s/m,d_{xy}=3.6\times 10^{-7}\;N\cdot s/m \end{array}$$

The reference trajectory is chosen to be $r_{1}=\sin(4.17t),r_{2}=1.2\sin(5.11t)$, close to its natural frequency in the x and y directions. Random variable signals with zero mean and unity variance plus sin(2πt) are selected as external disturbance d. Assume that the input angular velocity $\Omega_{z}=100\;\mathrm{rad}/s$. The reference length $q_{0}=1\;\mu m$. The reference frequency $w_{0}=1\;\mathrm{kHz}$. Simulation studies are implemented. The initial conditions are $q(0)={\left[\begin{array}{cc} 0 & 0 \end{array}\right]}^{T}$; the other parameters are selected as

$$\begin{array}{c} w_{1}=4.17,w_{2}=5.11,\tau =diag\{\begin{array}{ccccccc} 2 & 2 & 2 & 2 & 2 & 2 & 2 \end{array}\};\rho =diag\{\begin{array}{cc} 400 & 400 \end{array}\};\\ c=4;c_{1}=4;\hat{\theta }(0)=0.95\theta . \end{array}$$

**Figure 3:**
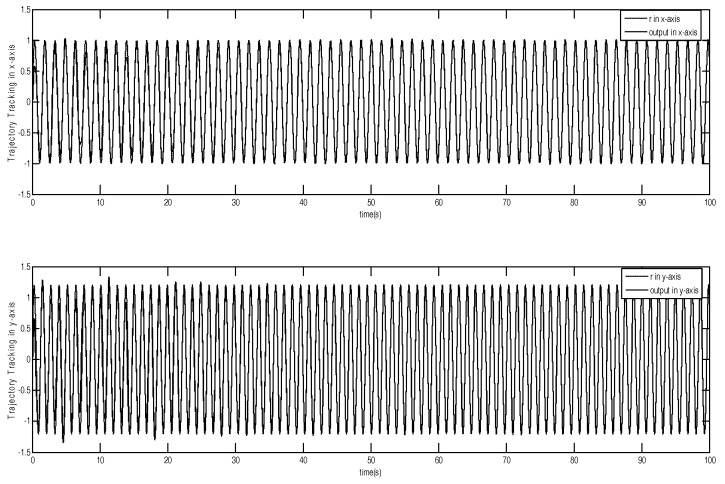
Trajectory tracking using adaptive dynamic sliding mode control based on the backstepping approach.

**Figure 4:**
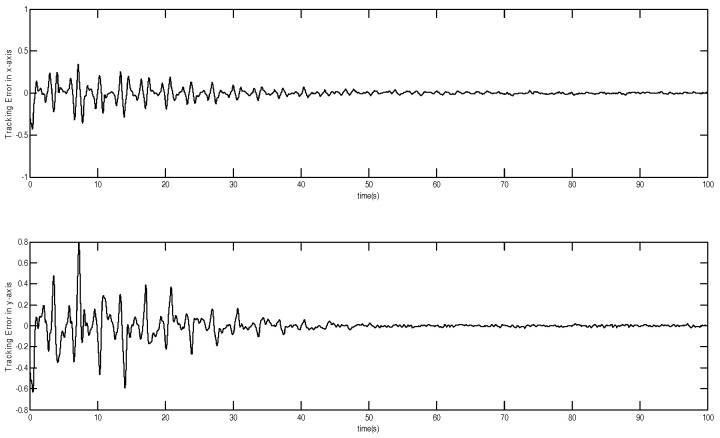
Tracking error using adaptive dynamic sliding mode control based on the backstepping approach.

**Figure 5:**
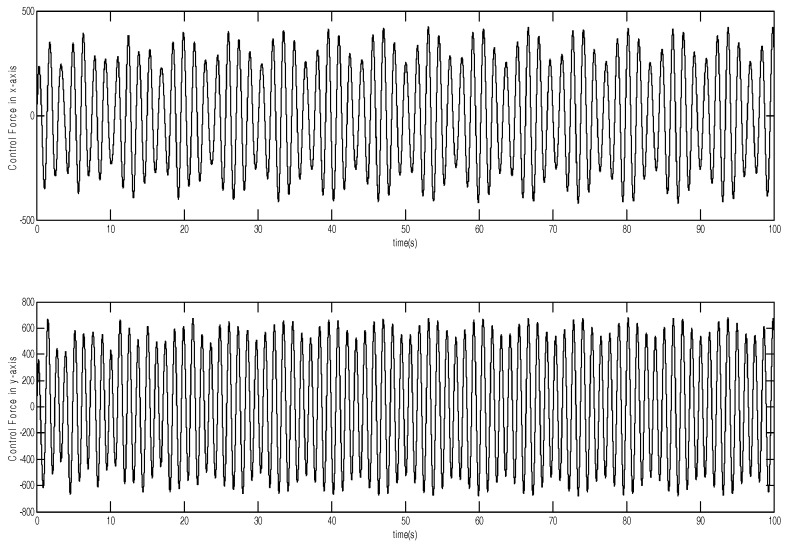
Control input using adaptive dynamic sliding mode control based on the backstepping approach.

**Figure 6:**
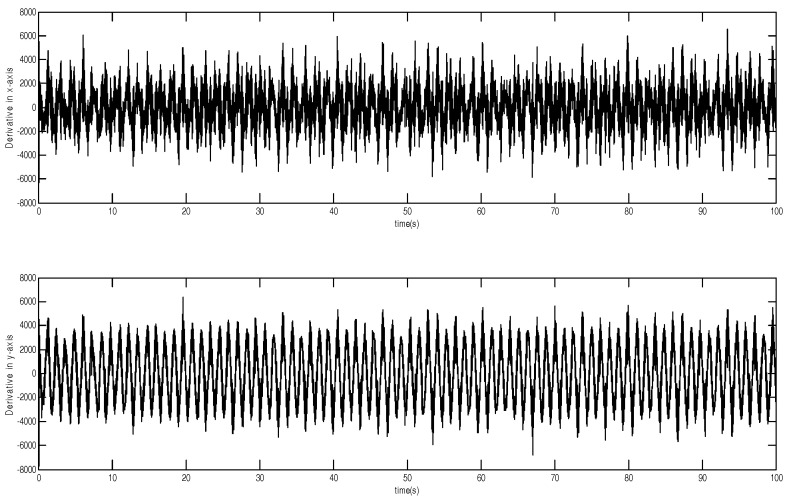
Control input derivative using adaptive dynamic sliding mode control based on the backstepping approach.

The tracking trajectory and tracking error are shown in Figure 3 and Figure 4. The control system can track the reference trajectory in 40 s. The control input and control input derivative using the adaptive DSMC method are drawn in Figure 5 and Figure 6, demonstrating that the adaptive DSMC with the backstepping design can transfer discontinuous terms to the first-order derivative of the control input, thereby decreasing the chattering.

**Figure 7:**
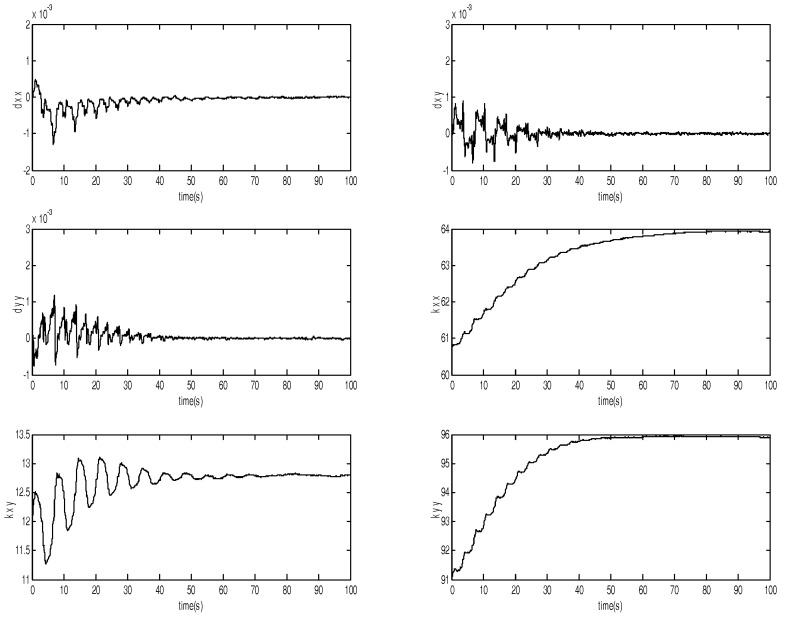
Parameter estimates of the MEMS gyroscope.

**Figure 8:**
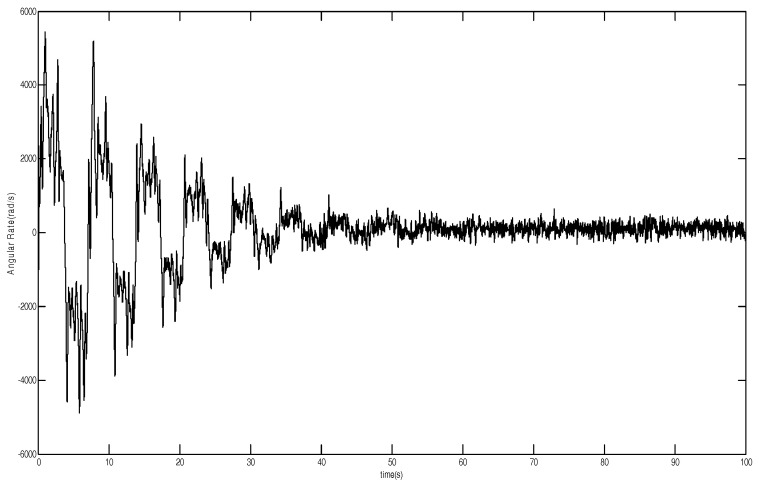
Convergence of angular velocity.

The parameters of the MEMS gyroscope are in Figure 7 and Figure 8, showing that the estimates of the spring and damping coefficients converge to their true values with a persistent sinusoidal reference signal. Therefore, the introduction of adaptive backstepping DSMC can adapt to the changing nonlinearities, which maintains the satisfactory performance. It means that DSMC not only removes some of the fundamental limitations of the traditional approach but also provides improved tracking accuracy.

## 5. Conclusions

In this study, an adaptive DSMC strategy with a backstepping approach was successfully applied to a MEMS gyroscope for the trajectory tracking. The derivative of the switching function is employed to differentiate classical sliding surface and transfer discontinuous terms to the first-order derivative of the control input, and effectively decrase the chattering. The asymptotical stability of the closed loop system can be guaranteed with the proposed DSMC strategy. Moreover, the proposed adaptive dynamic sliding mode control can estimate the system parameters online. Simulation studies are conducted to demonstrate the good performance of the proposed dynamic sliding mode control methods.
